# Spontaneous immunological activities in the target tissue of vitiligo-prone Smyth and vitiligo-susceptible Brown lines of chicken

**DOI:** 10.3389/fimmu.2024.1386727

**Published:** 2024-04-24

**Authors:** Daniel M. Falcon, Kristen A. Byrne, Marites A. Sales, Gisela F. Erf

**Affiliations:** Division of Agriculture, Department of Poultry Science, University of Arkansas System, Fayetteville, AR, United States

**Keywords:** autoimmune vitiligo, Smyth chicken, lymphocytes, cytokines, T cell receptor diversity, avian vitiligo model

## Abstract

**Introduction:**

Vitiligo is an acquired de-pigmentation disorder characterized by the post-natal loss of epidermal melanocytes (pigment-producing cells) resulting in the appearance of white patches in the skin. The Smyth chicken is the only model for vitiligo that shares all the characteristics of the human condition including: spontaneous post-natal loss of epidermal melanocytes, interactions between genetic, environmental and immunological factors, and associations with other autoimmune diseases. In addition, an avian model for vitiligo has the added benefit of an easily accessible target tissue (a growing feather) that allows for the repeated sampling of an individual and thus the continuous monitoring of local immune responses over time.

**Methods:**

Using a combination of flow cytometry and gene expression analyses, we sought to gain a comprehensive understanding of the initiating events leading to expression of vitiligo in growing feathers by monitoring the infiltration of leukocytes and concurrent immunological activities in the target tissue beginning prior to visual onset and continuing throughout disease development.

**Results:**

Here, we document a sequence of immunologically significant events, including characteristic rises in infiltrating B and αβ T cells as well as evidence of active leukocyte recruitment and cell-mediated immune activities (CCL19, IFNG, GZMA) leading up to visual vitiligo onset. Examination of growing feathers from vitiligo-susceptible Brown line chickens revealed anti-inflammatory immune activities which may be responsible for preventing vitiligo (IL10, CTLA4, FOXP3). Furthermore, we detected positive correlations between infiltrating T cells and changes in their T cell receptor diversity supporting a T cell-specific immune response.

**Conclusion:**

Collectively, these results further support the notion of cell-mediated immune destruction of epidermal melanocytes in the pulp of growing feathers and open new avenues of study in the vitiligo-prone Smyth and vitiligo-susceptible Brown line chickens.

## Introduction

Vitiligo is a common (0.5-1% worldwide population), acquired depigmentation disorder characterized by the progressive loss of pigment-producing cells (melanocytes) in the skin (1). Vitiligo is generally considered an autoimmune disorder with spontaneous disease onset suspected to result from a complex interaction of genetic, environmental, and immunological factors (2). Evidence of melanocyte-specific cell-mediated and humoral immune responses have been well documented in humans (3–9). Additionally, the use of immunosuppressive drugs is often prescribed either as a mono- or combination-therapy in an attempt to halt progression of the disease and re-stimulate pigmentation (10–12). Unfortunately, preventative measures are lacking due in part to the difficulty in collecting data before onset of the disease.

The Smyth line of chicken was originally described in 1977 and has since been well established as an excellent model for autoimmune vitiligo. Similarly to humans, Smyth chickens experience a spontaneous post-natal loss of epidermal melanocytes in the pulp of growing feathers through a complex interaction of genetic, environmental and immunological factors (13). In addition, the identification of an environmental trigger (vaccination of 1-day old chicks with live, cell-free turkey herpesvirus) has resulted in a reliable incidence of vitiligo (80-95%) among Smyth chicken hatch mates (14).

As mentioned, in chickens, epidermal melanocytes, the target cells in vitiligo, are located in growing feathers. The living portion of growing feathers (pulp) consists of a column of dermis surrounded by epithelial tissue (epidermis) which includes melanocytes and keratinocytes. The dermal and epidermal pulp tissue is generated from the dermal papilla and epidermal collar of the feather follicle, respectively, around which the connective tissue sheath is formed that encases the 8-10 mm column of the growing feather pulp tissue. At the proximal end (2-3 mm newest growth), melanocytes are located in an area of modified epidermis, which is subdivided into barb ridges. Each barb ridge is made up of epidermal melanocytes and columns of keratinocytes. The cell bodies of the epidermal melanocytes can be observed facing the dermis at the epidermal-dermal junction, forming a circle around the dermis, and their dendrites extend along the keratinocyte columns for melanosome transfer.

The growing feather can easily be removed (plucked) giving access to the enclosed, sterile target tissue (pulp) for laboratory analysis and allows for the continuous monitoring of local immune responses by sampling growing feathers of the same individual before and throughout development and progression of vitiligo. In fact, the growing feather is currently being utilized as a cutaneous test-tissue and bioassay to gain temporal insights into tissue/cellular responses to test materials injected into its dermis (15–17). With the easy, minimally invasive access to the target tissue, the Smyth line of chicken represents an ideal model to study the initiating events of spontaneously-occurring autoimmune vitiligo in growing feathers.

The full Smyth autoimmune vitiligo chicken model is composed of three lines of chicken: the vitiligo-prone Smyth chicken (incidence 80-95%), the vitiligo-susceptible, parental Brown line (incidence 0-2%) and the vitiligo-resistant, distantly related Light-brown Leghorn (incidence 0%) pigmentation-control line. The inherent genetic susceptibility to autoimmune vitiligo was revealed by intra-abdominal injection with the DNA methylation inhibitor 5-azacytidine. When treated in this manner, the incidence of vitiligo in Brown line chickens was over 70% compared to 0% in vehicle-injected chickens. In contrast there was no change in the incidence (0%) of vitiligo in Light-brown Leghorn chickens (18).

As in humans, the progression of vitiligo in Smyth chickens requires a functional immune system. This was demonstrated by the administration of corticosterone (an immunosuppressive steroid) which resulted in a significant reduction in vitiligo incidence compared to untreated controls (19). Furthermore, while evidence for a humoral response in Smyth chickens is well documented (20–23), it does not appear to be necessary for disease expression as removal of the bursa of Fabricius (site of B cell development in birds) resulted in a delay but not a prevention of vitiligo (24). Similar delays in onset were seen by the administration of cyclosporine A, an inhibitor of IL-2 release that has a marked effects on antigen-specific T cell proliferation (25).

While the exact roles of cell-mediated and humoral immunity in initiating autoimmune vitiligo are unclear, current evidence strongly argues for a T cell-driven etiology. In histological analysis of growing feather pulps from Smyth chickens with vitiligo, CD8^+^ T cells were found at the dermal/epidermal junction (anatomical location of melanocytes) in close proximity to apoptotic cells (26). Additionally, memory response-like leukocyte infiltration into wattles of completely depigmented Smyth chickens injected with feather-derived melanocyte lysates was dominated by T cells, although B cells were also detected (27). A microarray study examining pools of cDNA from growing feathers of Smyth chickens collected before and at disease onset found evidence of both humoral (Ig-J chain, CXCL13) and cell-mediated (CCL19, GZMA, IL21R) immune activities prior to vitiligo onset (28). More convincing evidence for a cell-mediated immune response came from a study examining the relationship between infiltrating leukocytes (estimated by immunohistochemical staining of frozen tissue sections) and cytokine gene expression profiles in growing feathers obtained prior to and throughout vitiligo progression. Elevated IFN-γ expression as well as a dominant presence of CD8^+^ cells leading up to visual onset, suggests a Th1-like, cell-mediated polarization in the active lesion (29).

The T cell compartment of a given individual is comprised of a variety (repertoire) of clones each with its own unique T cell receptor (TCR) capable of recognizing its own unique antigen displayed on MHC molecules of antigen-presenting cells. Upon its first encounter with an antigen-MHC complex, an activated T cell will undergo multiple rounds of proliferation, in essence skewing the repertoire. T cell receptors themselves are heterodimers with either an α- and β-chain or a γ- and a δ-chain. Antigen-specificity of the TCR is largely determined by a hypervariable region on the β and δ chains designated as the complementarity-determining region 3 (CDR3) which is generated by the random somatic recombination of varying alleles of germline-encoded variable (V), diversity (D) and joining (J) gene segments. The CDR3 itself results from the random addition and deletion of nucleotides at the junction of V-D and D-J gene segments (junctional diversity). The process yields T cell clone- and antigen-specific TCR β- and δ-chain genes with CDR3-regions that vary in their physical length. Therefore the distribution of lengths in a pool of recombined TCR β- and δ-chain genes can be used as a low resolution but fully comprehensive measure of T cell antigen-specificity diversity in the evolving autoimmune lesion (30–32).

Here, our objective was to gain a more comprehensive understanding of the relationship between infiltrating leukocytes and immunological activities in the evolving autoimmune lesion in growing feather pulps of Smyth chickens. To obtain individual profiles of pulp-infiltrating leukocytes and gene expression activities, sampling began before the onset of vitiligo and continued through complete depigmentation. Specifically, starting at 1-day post-hatch, growing feathers were sampled from individual chickens two times per week until day 113. Single cell suspensions were prepared from the entire pulp of growing feathers and the types and relative levels of pulp-infiltrating leukocytes were determined by immunofluorescent staining and flow cytometry. Gene expression profiles were also determined using the whole pulp and on the level of the individual. Lastly, changes in the overall diversity of the T cell repertoire in the evolving autoimmune lesion were examined. Separate individual profiles of all parameters were also obtained for a parental control, the vitiligo-susceptible but non-expressing Brown line chickens. Results from this study will not only provide population-level insights into the initiating events of autoimmune vitiligo in Smyth chickens but will also allow for the study of the homogeneity of the response using data at the level of the individual.

## Materials and methods

### Animal care

All chickens used in this study were from the Smyth and Brown line populations maintained by G. F. Erf at the University of Arkansas System, Division of Agriculture (UADA), Poultry Research Farm in Fayetteville, AR. Fourteen vitiligo-prone Smyth and six MHC-matched (*B* ^101/101^) parental-control Brown line chicks (straight-run) were randomly selected from a replacement hatch for the study. All chicks were vaccinated against Marek’s disease with live herpesvirus of turkey (Fort Dodge Animal Health, Fort Dodge, IA) at day of hatch (day 1) and kept in floor pens on wood shavings with free access to food and water at the UADA Poultry Research Farm. All studies involving animals were conducted with the approval of the University of Arkansas Institutional Animal Care and Use Committee (IACUC) as outlined in protocol 15015.

### Vitiligo scoring and feather sampling

Prior to sampling, all chickens were visually scored for signs of depigmentation (indicative of vitiligo onset). Specifically, scoring was done by close examination of the most proximal end (newest growth) of growing feather shafts for signs of depigmentation. Both scoring and feather sampling began at 1-day post-hatch and continued twice a week until day 113 (approximately 16 weeks). Pulp-containing growing feather samples taken during days 1-26 were plucked from the wing and thereafter (days 29-113) from the breast tract. For each chicken and each time point, three growing feather samples were taken with one being placed in D-PBS on ice, and the other two in Tissue-Tek® O.C.T. Compound (Sakura of America, Hayward, CA) and snap-frozen in liquid nitrogen. Samples stored in D-PBS were used for same-day population analysis of pulp infiltrating leukocytes (see below). Samples snap-frozen in O.C.T. were stored at -80°C until use.

### Population analysis of infiltrating leukocytes

Single-cell suspensions were obtained from the feather pulp as previously described (15). Briefly, a longitudinal slit was made along the feather sheath and the pulp was removed, placed in a D-PBS solution containing 0.1% collagenase (type IV, Life Technologies, Carlsbad, CA) and 0.1% dispase II (Boehringer Mannheim, Mannheim, Germany) and incubated for one hour at 40°C. Infiltrating cells were liberated from the digested pulp tissue by passage through a 60 µm nylon mesh and washed in a D-PBS solution containing 1% bovine serum albumin (VWR, Randor, PA) and 0.1% sodium azide (VWR, Randor, PA). Leukocyte populations in prepared single-cell suspensions were immunofluorescently stained using the following chicken-specific mouse-monoclonal antibodies: total leukocytes (CD45-SPRD), macrophages (KUL01-rPE), B cells (Bu-1-FITC), γδ T cells (TCR1-FITC), αβ_1_ T cells (TCR2-FITC), αβ_2_ T cells (TCR3-rPE), T-helper cells (CD4-FITC) and cytotoxic T cells (CD8α-rPE) (**Supplementary Table 1**). To control for non-specific binding and identify negative populations for gating, a pool of all samples was prepared and incubated with a cocktail of mouse IgG1 isotype-control antibodies (FITC-, rPE- and SPRD-conjugated). Pooled samples were also single-stained with anti-CD45-FITC, -rPE and –SPRD antibodies in order to set compensation. Stained samples were acquired (10,000 events) on a Becton Dickinson FACSort flow cytometer equipped with a 488nm laser. Data analysis was performed using FlowJo software (FlowJo, LLC, Ashland, OR). Forward- versus side-scatter dot plots were used to filter out debris and data were expressed as the percentage of all acquired events (% pulp cells). Gating strategies are provided in **Supplementary Figure 1**.

### RNA extraction and cDNA synthesis

Frozen feathers sampled from 1d – 61d were processed for RNA extraction. To ensure complete removal of O.C.T., frozen feather samples were thawed in 70% ethanol. Once thawed the pulp was removed as above and immediately placed in TRI Reagent® (Zymo Research, Irvine, CA). Extracted pulp tissue was homogenized in TRI Reagent using a Tissue-Tearor™ (BioSpec Products, Bartlesville, OK, model# 985370-395) and total RNA was isolated using the Direct-zol™ RNA Miniprep kit (Zymo Research, Irvine, CA) with on-column DNase digestion according to the manufacturer’s instructions. RNA was eluted in 30µL DEPC-treated molecular-grade water (VWR, Radnor, PA) and quantity and purity was assessed by absorbance at 260nm and 280nm on a BioTek Synergy HT (Winooski, VT). One microgram of RNA was reverse transcribed to cDNA in a 20µL sample volume (50ng/µL) using the High Capacity cDNA Reverse Transcription kit (Applied Biosystems, Foster City, CA) according to the manufacturer’s instructions. Synthesized cDNA was diluted to 10ng/µL using DEPC-free molecular-grade water (VWR, Radnor, PA) and stored at -20°C until use.

### Gene expression analysis

Quantitative real-time PCR was performed as previously described using the TaqMan™ system with modifications (33). Target primer and probe sequences are listed in **Supplementary Table 2**. Reactions were conducted in a 12µL sample volume using 20ng of cDNA and were run on an Applied Biosystems® 7500 Real-Time PCR System using manufacturer-programmed cycling conditions. Relative gene expression was determined by the efficiency-calibrated ΔΔCt method (34) and is expressed as fold change relative to the calibrator sample. To obtain gene expression profiles for individual chickens, results were calculated using individual-specific day 29 samples as calibrators and 28S as a reference gene.

### Amplification of CDR3-containing regions of T cell receptor β-chains

Complementarity-determining region 3 (CDR3)-containing regions of the T cell receptor-β_1_ (TCR-β_1_) and –β_2_ (TCR-β_2_) chain genes were amplified from individual cDNA samples (prepared above) by end-point PCR. Using all available coding sequences in the NCBI database, fluorescently-labeled (forward) primers were designed against conserved 5’-variable (V) gene segments of TCR-β_1_ (TCR-V_β_1) and TCR-β_2_ (TCR-V_β_2) genes. An unlabeled (reverse) primer was designed in a similar manner against a conserved 3’-constant (C) region shared by the two genes. Specificity of TCR-V_β_1 and TCR-V_β_2 forward primers was confirmed by testing against cDNA derived from TCR1 (γδ T)-, TCR2 (αβ_1_ T)- and TCR3 (αβ_2_ T)-sorted PBMCs. Primer sequences and labels are listed in **Supplementary Table 3**. Reactions were conducted in a 20µL sample volume using 20ng cDNA, 0.5µM primers and Phusion™ High-Fidelity PCR Master Mix (Thermo Scientific, Waltham, MA). Cycling conditions were as follows: 98°C for 20s (initial denaturation), 98°C for 10s (denaturation), 60°C for 30s (annealing), 72°C for 30s (extension), 72°C for 5 min (final extension) and held at 4°C.

### Quantification of changes in the T cell receptor repertoire

Fluorescently-labeled PCR products amplified from individual cDNA samples were sent to the DNA Analysis Facility on Science Hill at Yale University (New Haven, CT) for size-separation by capillary electrophoresis on a 3730xl DNA Analyzer (Applied Biosystems, Foster City, CA). Analysis of raw spectrographs (fluorescence signal versus size) for each sample was performed using GeneMapper® 4.0 (Applied Biosystems, Foster City, CA) with fluorescence height as the measure of quantity. To discount non-specific fluctuations in the fluorescence signal, initial peak detection was performed using thresholds of 100 and 50 for TCR-V_β_1 and -V_β_2 samples respectively. Alleles were defined based on an overlay of all spectrographs and were separated by approximately three base pairs. Each individual CDR3 length profile was then converted to a frequency distribution by dividing the fluorescence height of a specific allele by the total fluorescence of all alleles in the sample. To quantify overall changes in CDR3 frequency distributions over time the Hamming-distance (D-score) was calculated against a reference sample. Specifically, the absolute values of the difference in frequencies for every allele relative to samples taken at 1 day of age were summed and then divided by 2 (theoretical minimum and maximum: 0% and 100%, respectively (31).

### Statistical analysis

Prior to statistical testing, gene expression (fold change) data were converted from an exponential to a linear scale by log_2_ transformation. No transformation was required for leukocyte infiltration (% pulp cells) or T cell receptor diversity data (D-score). Data for vitiligo-expressing Smyth chickens were aligned and averaged according to time (days) with respect to vitiligo onset (set to 0 d). Data for non-expressing Brown line chickens were reported and averaged based on age (days). To determine the trend with respect to time on all measurements, a mixed-effects regression model was used. Time was identified as a fixed effect and individual chickens as a random effect using a residual covariance structure. When a significant effect was found, post-hoc multiple means comparisons were made against the -39 d sample using the Tukey-Kramer p-value adjustment. Correlation analysis was done using Spearman’s method. Differences were considered significant when *P* ≤ 0.05. All statistical analyses were done using JMP Pro 13 (SAS Institute Inc., Cary, NC).

## Results

### Vitiligo incidence

At the conclusion of the study, 10 Smyth chickens had developed vitiligo. One Smyth chicken with no signs of vitiligo died at 26 days and was replaced with another chicken, also with no signs of vitiligo, at the following time point (29 days). Two Smyth and one Brown line chicken had signs of sporadic progression of vitiligo, however, only samples from Smyth chickens which had progressed from fully pigmented to fully depigmented (n = 8) and Brown line chickens that remained fully pigmented (n = 5) were selected for further analysis. As expected, individual Smyth chickens developed vitiligo at different ages: two at 43 days, three at 47 days, one at 54 days and two at 57 days.

### Infiltration of leukocytes into growing feathers of Smyth chickens relative to vitiligo onset

For statistical analysis, data points for vitiligo-expressing Smyth chickens were aligned and averaged with respect to time (days) of vitiligo onset (set to 0 d). In order to increase the power of statistical testing, time points with less than 3 data points were excluded which resulted in an analysis range of 39 days prior- through 68 days post-vitiligo onset (-39 d to 68 d). In order to determine periods of significant infiltration, multiple means comparisons were made against the -39 d time point.

**Figure 1:**
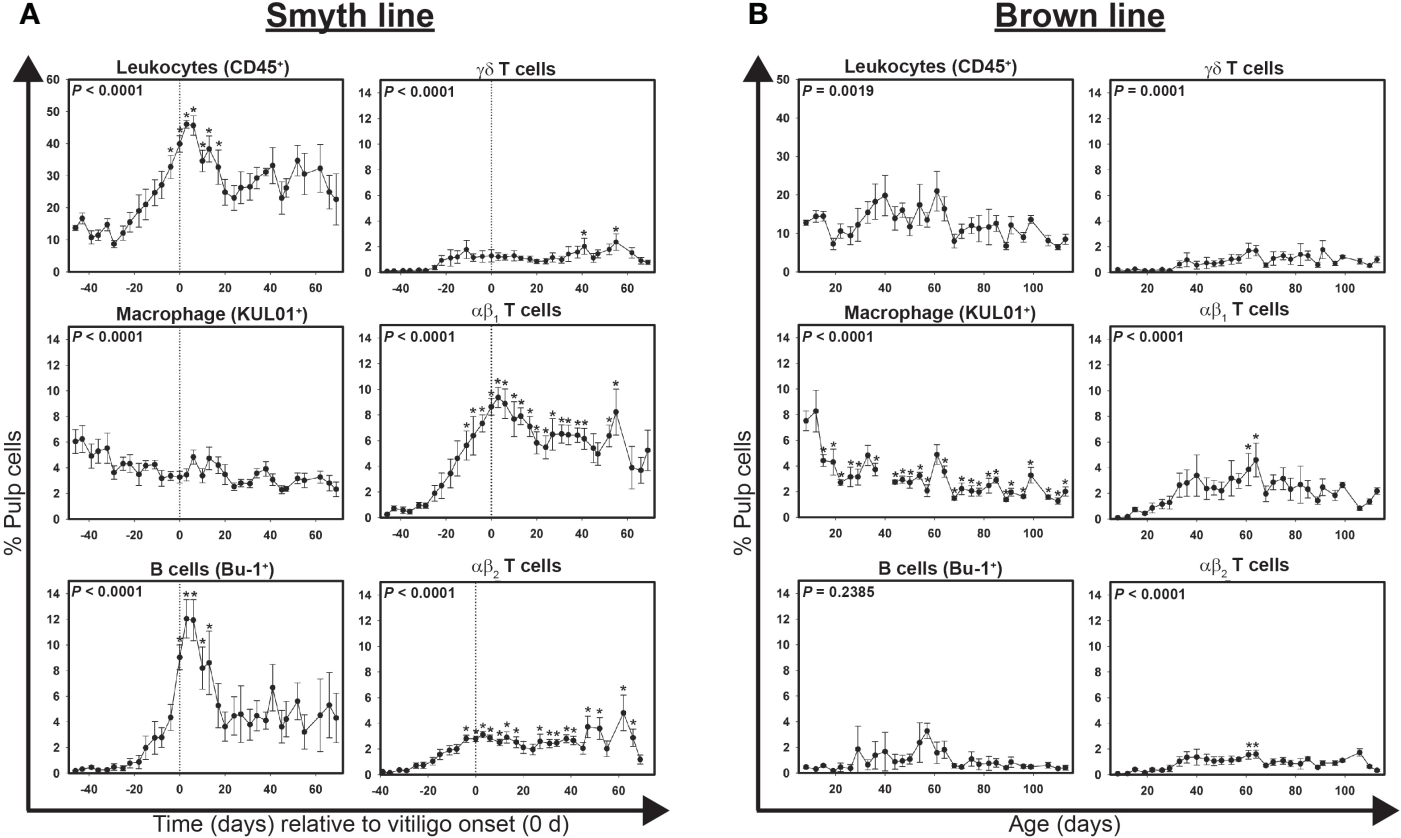
Cell infiltration profiles for total leukocytes, macrophages, B cells and T cells in growing feathers of vitiligo-expressing Smyth and non-expressing Brown line chickens. Growing feathers were sampled beginning at 1-day post-hatch and continued twice a week for 113 days. Single-cell suspensions were prepared from feather samples and immunofluorescently-stained for total leukocytes (CD45), macrophages (KUL01), B cells (Bu-1), γδ T cells (TCR1), αβ1 T cells (TCR2) and αβ2 T cells (TCR3) using mouse monoclonal chicken-specific antibodies. Population analysis was performed by flow cytometry. **(A)** Data for vitiligo-expressing Smyth chickens were aligned by time (days) relative to age of vitiligo onset (set to 0 d and indicated by vertical dashed line). **(B)** Data for non-expressing Brown line chickens were aligned and averaged by age (days). Smyth chickens were 43 to 57 days of age at time of vitiligo onset. For both Smyth and Brown line data, the overall effect of time was determined using a mixed effects regression model setting onset time (A)/age (B) and individual bird as a fixed and random effect, respectively. When a significant time effect was found, post-hoc multiple means comparisons were made against the -39 d or 8 d sample for Smyth or Brown line data, respectively, using the Tukey-Kramer p-value adjustment. Differences were considered significant at *P* < 0.05 (*). Data are plotted as mean ± SEM; n = 3 to 8 chickens per time point.

Highly significant (*P* < 0.0001) changes in total leukocytes (CD45^+^), macrophages (KUL01^+^), B cells (Bu-1^+^), γδ T cells (TCR1^+^), αβ_1_ T cells (TCR2^+^) and αβ_2_ T cells (TCR3^+^) levels were detected over time in growing feathers of vitiligo-expressing Smyth chickens (**Figure 1A**). Baseline (-39 d) levels of total leukocytes averaged 10.8 ± 2.1% pulp cells. Two weeks prior to visual onset (-15 d) leukocyte levels were roughly double (21 ± 4.8% pulp cells) and by -4 d had risen to statistical significance (32.8 ± 3.5% pulp cells; *P* < 0.05) reaching a maximum of 46.0 ± 1.2% pulp cells (*P* < 0.05) 3 days post visual onset (3 d). Total leukocyte levels remained statistically elevated until 17 days post onset (32.3 ± 7.5% pulp cells; *P* < 0.05) at which point they lowered to roughly double the baseline levels (~20% pulp cells).

At -39 d, macrophages accounted for over 45% of total leukocytes (4.9 ± 0.9% pulp cells), but by visual onset (0d) had dropped down to 8.2% of total leukocytes (3.3 ± 0.5% pulp cells). While no significant differences relative to the baseline (4.9 ± 0.9% pulp cells) were found, the percentage of macrophages in the growing feather continued to decline throughout complete depigmentation (*P* < 0.001).

At -39 d, B cell levels were a mere 0.5 ± 0.1% pulp cells, however by visual onset their levels increased to 9.0 ± 1.0% pulp cells, having more than doubled from the previous time point (-4 d; 4.4 ± 1.0; *P* < 0.05). After reaching a maximum of 11.9 ± 1.6% pulp cell 6 days post onset (*P* < 0.05), B cells, while still numerically above baseline, declined to non-significant levels.

Significant changes in γδ T cells were not detected until 41 and 55 days post visual onset (2.0 ± 0.6 and 2.4 ± 0.6% pulp cells, respectively; *P* < 0.05). At -39 d, γδ, αβ_1_ and αβ_2_ T cells measured 0.1 ± 0.0, 0.6 ± 0.3 and 0.2 ± 0.1% pulp cells respectively. Significant infiltration of αβ_1_ T cells were detected as early as -11 d (5.6 ± 1.1% pulp cells; *P* < 0.05) and remained statistically elevated until 41 d (6.1 ± 0.8% pulp cells; *P* < 0.05) having reached maximum levels at 3 d (9.4 ± 0.8% pulp cells; *P <* 0.05). In contrast, statistically elevated levels of αβ_2_ T cells were not seen until -4 d (1.6 ± 0.4% pulp cells; *P* < 0.05) and did not reach their maximum until 62 days post onset (4.8 ± 1.4% pulp cells; *P* < 0.05).

### Infiltration of leukocytes into growing feathers of non-vitiligo-expressing Brown line chickens

Data points for Brown line controls (n = 5) were aligned and averaged by age (days) with multiple means comparisons being made against the sample collected at 8 days of age. Highly significant changes in total leukocytes (*P* = 0.0019), macrophages (*P* < 0.0001), γδ T (*P* = 0.0001), αβ_1_ T (*P* < 0.0001) and αβ_2_ T cells (*P* < 0.0001) were also observed in growing feathers of non-expressing Brown line control chickens (**Figure 1B**). In contrast to levels in growing feathers of Smyth chickens, total leukocytes never rose above 21% total pulp cells and there were no significant increases found relative to 8 d samples. Similar to Smyth chickens, macrophage levels steadily declined over the course of the study. In particular between 12 d and 15 d of age macrophages level dropped from 8.3 ± 1.6 to 4.4 ± 0.5% pulp cells (*P* < 0.05). Interestingly, a transient elevation in αβ_1_ and αβ_2_ T cell levels was observed at 61 d (3.9 ± 1.3 and 1.6 ± 0.3% pulp cells, respectively; *P* < 0.05) and 64 d (4.6 ± 1.3 and 1.6 ± 0.3% pulp cells, respectively). In complete contrast to Smyth chickens, no increases in B cells were observed in growing feathers from Brown line chickens (*P* = 0.2385).

### Infiltration of CD4^+^, CD8α^+^ and CD4^+^CD8a^+^ cells in growing feathers of Smyth chickens

**Figure 2:**
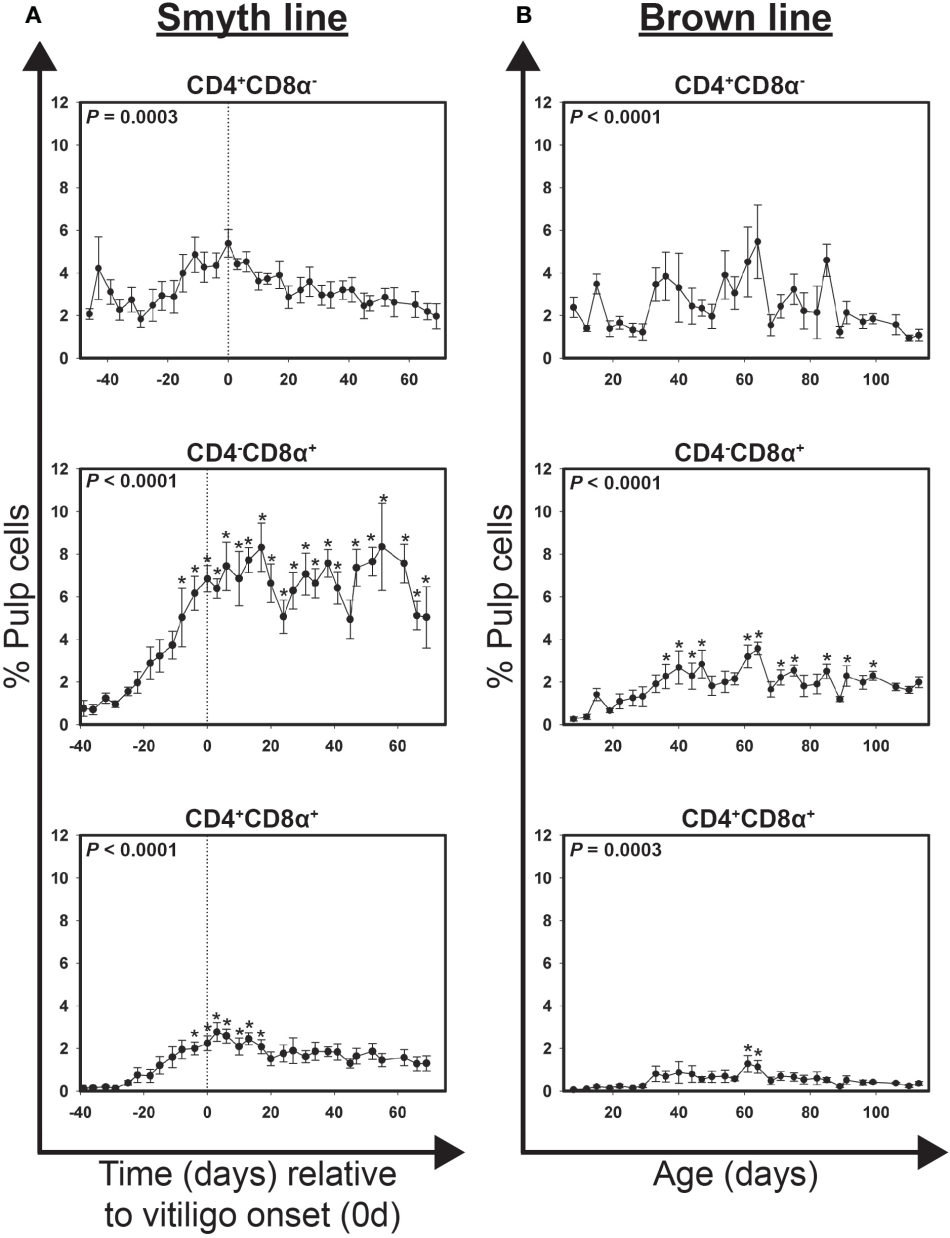
Cell infiltration profiles for T cell subsets in growing feathers of vitiligo-expressing Smyth chickens. Growing feathers were sampled from Smyth chickens beginning at 1-day post-hatch and continued twice a week for 113 days. Single-cell suspensions were prepared from feather samples and immunofluorescently-stained for CD4 and CD8α using mouse monoclonal chicken-specific antibodies. Population analysis was performed by flow cytometry. **(A)** Data for vitiligo-expressing Smyth chickens were aligned by time (days) relative to age of vitiligo onset (set to 0 d and indicated by vertical dashed line). Smyth chickens were 43 to 57 days of age at time of vitiligo onset. **(B)** Data for non-expressing Brown line chickens were aligned and averaged by age (days). For both Smyth and Brown line data, the overall effect of time was determined using a mixed effects regression model setting onset time (A)/age (B) and individual bird as a fixed and random effect, respectively. When a significant time effect was found, post-hoc multiple means comparisons were made against the -39 d or 8 d sample for Smyth or Brown line data, respectively, using the Tukey-Kramer p-value adjustment. Differences were considered significant at *P* < 0.05 (*). Data are plotted as mean ± SEM; n = 3 to 8 chickens per time point.

Highly significant changes in the levels of CD4^+^CD8α^-^, CD4^-^CD8α^+^ and CD4^+^CD8α^+^ T cells were detected over time in growing feathers of vitiligo-expressing Smyth chickens (*P* = 0.0003, < 0.0001 and < 0.0001, respectively; **Figure 2A**). At -39 d CD4^+^CD8α^-^, CD4^-^CD8α^+^ and CD4^+^CD8α^+^ cells accounted for 3.1 ± 0.6, 0.8 ± 0.4 and 0.1 ± 0.0% pulp cells, respectively. CD4^-^CD8α^+^ cells were significantly elevated as early as -8d (5.0 ± 1.4% pulp cells; *P* < 0.05) and reached their maximum at 17 d and 55 d (8.3 ± 0.6 and 8.3 ± 2.0% pulp cells, respectively; *P* < 0.05). Interestingly, while CD4^+^CD8α^-^ cells reached their maximum level at onset (0 d; 5.4 ± 0.7% pulp cells), these increases were not statistically significant. CD4^+^CD8α^+^ cells (phenotypically CD8α-low) were transiently elevated from -4 d (2.0 ± 0.3% pulp cells; *P* < 0.05) to 17 d (2.1 ± 0.3% pulp cells; *P* < 0.05).

### Infiltration of CD4^+^, CD8α^+^ and CD4^+^CD8α^+^ cells in growing feathers of parental-control, non-expressing Brown line chickens.

Highly significant changes in the levels of CD4^+^CD8α^-^, CD4^-^CD8α^+^ and CD4^+^CD8α^+^ T cells were also detected in growing feathers of Brown line chickens (*P* < 0.0001, < 0.0001 and = 0.0003, respectively; **Figure 2B**). Transient increases in CD4^-^CD8α^+^ were seen from 36 d – 47 d (*P* < 0.05) and again from 61 d – 64 d (*P* < 0.05) though levels never rose above 4% of pulp cells. A similar increase from 61 d – 64 d (*P* < 0.05) was found in CD4^+^CD8α^+^ cells though levels never rose above 1.5% of pulp cells. Similar to Smyth chickens, no significant differences in the levels of CD4^+^CD8α^-^ cells were found relative to the 8 d of age samples.

### Early markers of immunological activities in growing feathers of Smyth chickens relative to vitiligo onset

**Figure 3:**
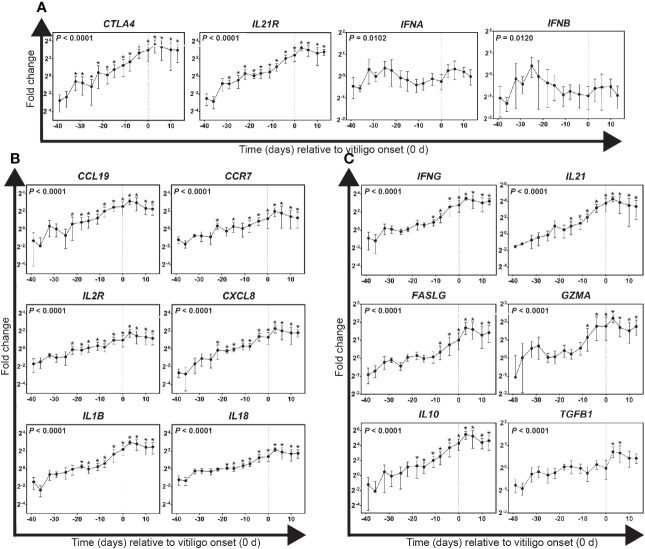
Gene expression profiles for immunological activities in growing feathers of vitiligo-expressing Smyth chickens. Growing feather samples were taken from Smyth chickens beginning at 1-day post-hatch and continuing twice a week for 113 days. cDNA from samples collected from 39 days before- through 13 days post-vitiligo onset (i.e. -39 d to 13 d) were analyzed for relative gene expression of: **(A)** potential early markers of immunological activities, **(B)** markers of pro-inflammatory and recruitment activities and **(C)** markers of cell-mediated immunity and anti-inflammatory activities. Relative gene expression was determined by the efficiency-calibrated ΔΔCt method and is expressed as fold change relative to the calibrator sample. Gene expression profiles were obtained for individual chickens, using individual-specific day 29 samples as calibrators and 28S as a reference gene. Data were aligned and averaged by time (days) relative to age of vitiligo onset (set to 0 d; indicated by vertical dashed line). Smyth chickens were 43 to 57 days of age at time of vitiligo onset. The overall effect of time was determined using a mixed effects regression model setting time and individual bird as a fixed and random effect respectively. When a significant time effect was found, post-hoc multiple means comparisons were made against the -39 d samples using the Tukey-Kramer p-value adjustment. Differences were considered significant at *P* < 0.05 (*). Data are plotted as mean ± SEM; n = 3 to 8 chickens per time point.

Targeted gene expression was conducted on cDNA prepared from samples collected between 45 days before and 13 days post-vitiligo onset from Smyth chickens. Highly significant changes in expression of *CTLA4* and *IL21R* were found in growing feathers of vitiligo-expressing Smyth chickens (*P* < 0.0001; **Figure 3A**). Both genes demonstrated a similar trend of a steady increase leading up to vitiligo onset (0 d), reaching peak levels 3 days post-onset and maintaining a sustained elevation thereafter. The earliest signs of immunological activity were in the significant increased expression of the anti-inflammatory co-receptor CLTA4 (-32 d; *P* < 0.05) with the significant increase in *IL21R* expression seen three days later (-29 d; *P* < 0.05). Interestingly, while significant changes in overall gene expression were found for type-1 interferons (*IFNA*, *P* = 0.1020; *IFNB*, *P* = 0.0120) no differences were found relative to -39 d samples.

### Markers of pro-inflammatory and recruitment activities in growing feathers of Smyth chickens relative to vitiligo onset

Highly significant changes in the expression of recruitment and homing-related genes *CCL19*, *CCR7* and *CXCL8* were found in growing feathers of vitiligo-expressing Smyth chickens (*P* < 0.0001; **Figure 3B**). Initial increases for all four genes began 22 days before visual onset (*P* < 0.05) after which levels continued to rise reaching maximum expression 3 days post-onset and remained elevated thereafter. Highly significant changes in the expression of pro-inflammatory genes *IL1B* and *IL18* (*P* < 0.0001) were also found (**Figure 3B**). Beginning 18 days before visual onset, levels of *IL1B* and *IL18* were significantly increased and similar to the chemokine genes, reached maximum expression 3 days post onset, and remained elevated thereafter.

### Markers of T cell activation and anti-inflammatory activities in growing feathers of Smyth chickens relative to vitiligo onset

Highly significant changes in the expression of T cell activation-related genes *IFNG*, *FASLG*, *GZMA* and *IL21* and anti-inflammatory genes *IL10* and *TGFB1* were found in growing feathers of vitiligo-expressing Smyth chickens (*P* < 0.0001; **Figure 3C**). All measured gene expression demonstrated a similar trend upwards leading up to vitiligo onset reaching maximum levels 3 days post onset and maintaining a sustained elevation thereafter. Interestingly, significant expression of the anti-inflammatory cytokine *IL10* preceded significant elevation of T cell activation markers. Eighteen days prior to visual onset saw the first significant increase in *IL10* (*P* < 0.05) followed by significant elevation of *IL21*, a cytokine responsible for the maintenance of activated T cells, three days later (-15 d, *P* < 0.05). *IFNG*, the signature cytokine of a cell-mediated immune response was significantly elevated starting at -11d followed by increases in cytotoxic T cell markers *FASLG* and *GZMA* three days later (-8 d, *P* < 0.05). Lastly, significant expression of the anti-inflammatory cytokine *TGFB1* was seen at 3 d and 6 d (*P* < 0.05).

### Alterations in the T cell repertoire in growing feathers precede visual onset of vitiligo in Smyth chickens

**Figure 4:**
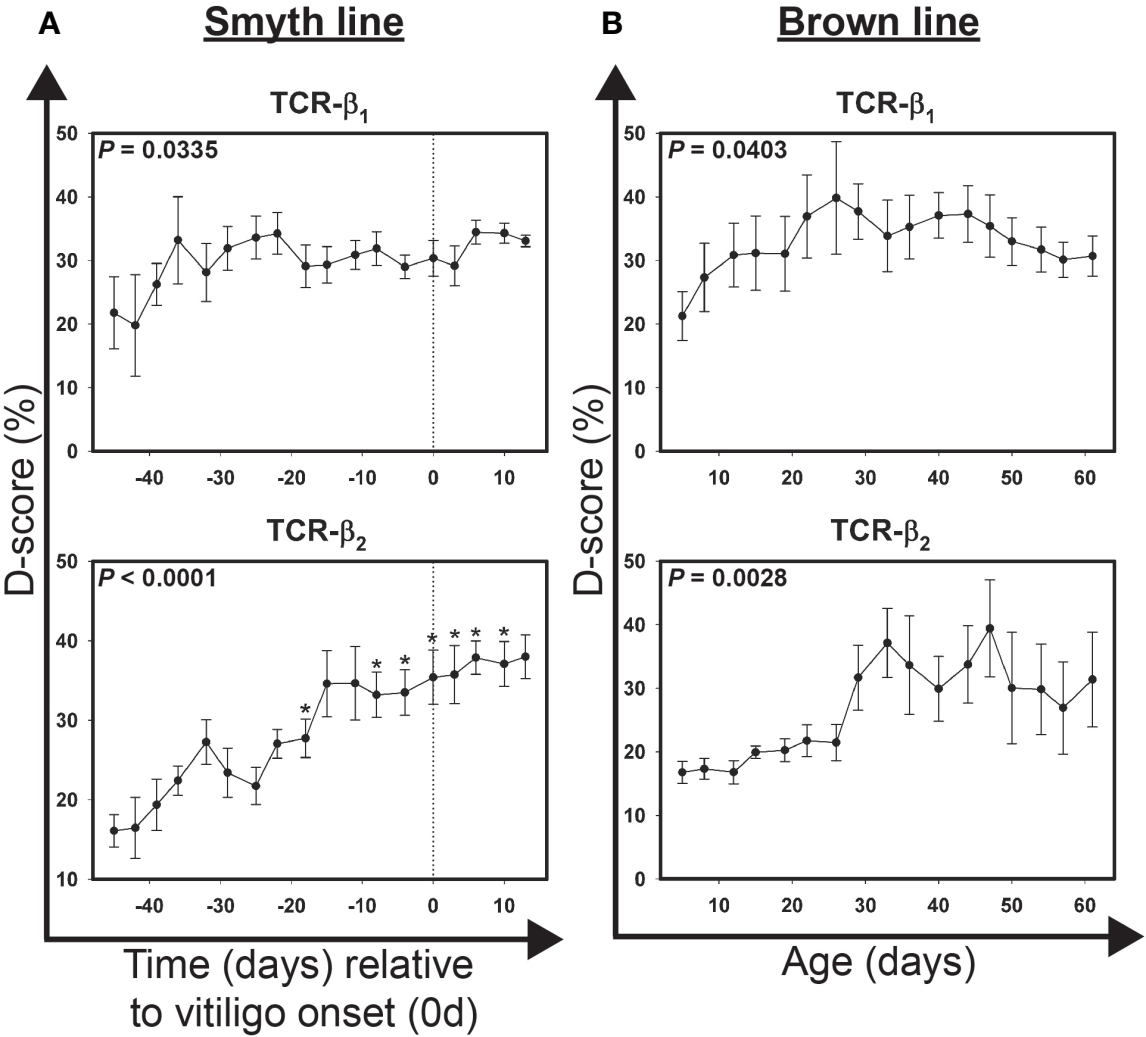
Alterations in the T cell receptor repertoire in growing feathers of vitiligo-expressing Smyth chickens and non-vitiligo expressing Brown line chickens. (**A**) Growing feathers were sampled from Smyth beginning at 1-day post-hatch and continued twice a week for 113 days. Complementarity-determining 3 (CDR3)-containing regions of the T cell receptor-variable β1 (TCR-β1) and -variable β2 (TCR-β2) domain genes were amplified from cDNA derived from growing feather samples by end-point PCR using fluorescently-labeled forward primers specific to conserved 5’ regions of variable (V) gene segments and an unlabeled reverse primer specific to conserved 3’ constant regions shared by the two genes. The resulting fluorescently-labeled products were size-separated by capillary electrophoresis and the quantities of each group of alleles estimated based on fluorescence intensity. Quantification profiles for each sample were converted to a frequency distribution and the Hamming-distance (D-score) was calculated by summing the absolute difference of each allele group relative to a reference sample and then dividing by 2. cDNA from samples collected from 45 days before- through 13 days post-vitiligo onset (i.e. -39 d to 13 d) were analyzed for alterations in the T cell receptor repertoire. Data were aligned by time (days) relative to age of vitiligo onset (set to 0 d). Smyth chickens were 43 to 57 days of age at time of vitiligo onset. **(B)** cDNA from samples collected from 5 days through 61 days of age were analyzed for alterations in the T cell receptor repertoire. Data for Brown line controls were aligned and averaged by age (days). The overall effect of time was determined using a mixed effects regression model setting time and individual bird as a fixed and random effect respectively. When a significant time effect was found, post-hoc multiple means comparisons were made against the -39 d using the Tukey-Kramer p-value adjustment. Differences were considered significant at *P* < 0.05 (*). Data are plotted as mean ± SEM; n = 3 to 8 per time point.

To examine overall changes in the diversity within the TCR repertoire (D-score) with respect to vitiligo onset in growing feathers of Smyth chickens, cDNA from samples collected between 45 days before and 13 days post-onset were used (**Figure 4A**). D-scores for TCR-β1 ranged from 23.1 ± 4.2% (-45 d) to 36.8 ± 6.6% (-36 d) (*P* = 0.0335), however no sample differed significantly from -39 d. In contrast D-scores for TCR-β2 ranged from 14.5 ± 2.2% (-45d) to 38.5 ± 2.4% (10d) (*P* < 0.0001) with significant overall changes seen at -18 d (35.6 ± 4.0%; *P* < 0.05) and -8 d through 10 d (*P* < 0.05).

### Alterations in the T cell repertoire in growing feathers of non-expressing Brown line chickens

To examine overall changes in the diversity within the TCR repertoire (D-score) with respect to age in growing feathers of Brown line chickens, cDNA from samples collected between ages 5 d and 61 d were used (**Figure 4B**). D-scores for TCR-β1 ranged from 21.3 ± 3.8% (5 d) to 39.8 ± 8.9% (26 d) (*P* = 0.0403). Significant changes in TCR-β2 diversity were also found (*P* = 0.0028). D-scores ranged from 16.8 ± 1.7% (5 d) to 39.4 ± 7.6% (47 d), however no sample differed significantly from 5 day samples. Interestingly, variability in TCR-β2 D-score profile in individual Brown line chickens increased substantially after 26 d of age.

### Association of overall changes in the TCR repertoire with infiltrating T cell subsets in growing feathers of Smyth chickens

To determine whether increases in overall changes in the T cell receptor repertoire (D-score) were associated with levels of T cells and their subsets in growing feather samples from vitiligo-expressing Smyth chickens, D-scores and pulp-infiltration data were tested for correlation by Spearman’s method (**Supplementary Table 4**). Highly significant, positive correlations between the D-score of TCR-β2 and infiltrating αβ_2_ T (*P* < 0.0001), CD4^-^CD8α^+^ (*P* < 0.0001) and CD4^+^CD8α^+^ (*P* = 0.0003) cells were found in data obtained from vitiligo-expressing Smyth chickens. No significant correlations between TCR-β1 D-scores and infiltrating T cells were found in samples from Smyth chickens.

### Expression of recruitment and anti-inflammatory genes in growing feathers from Brown line chickens

**Figure 5:**
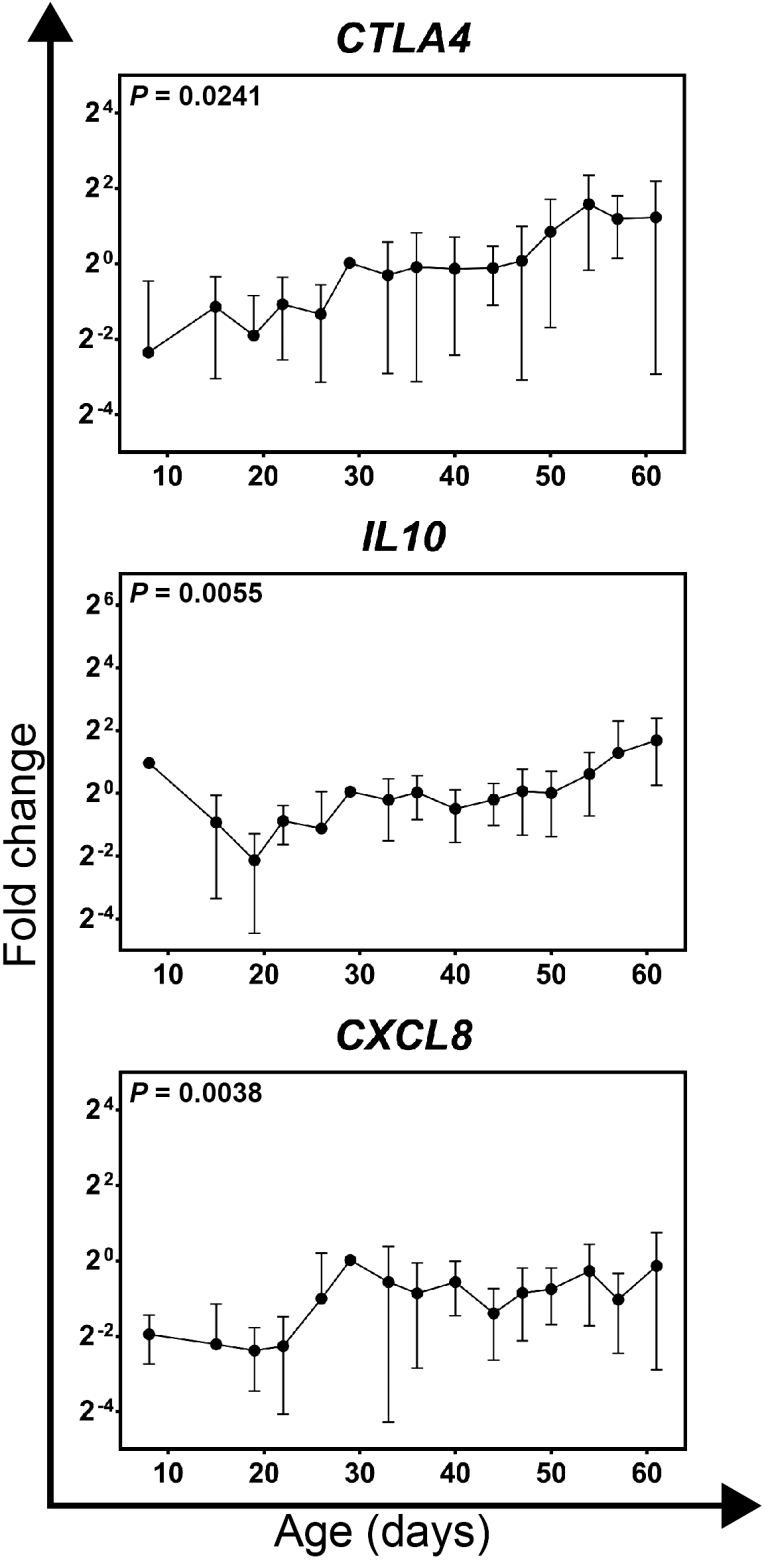
Gene expression profiles for genes significantly affected by age in growing feathers of parental-control, non-vitiligo expressing Brown line chickens. Growing feather samples were taken from Brown line chickens beginning at 1-day post-hatch and continuing twice a week for 113 days. cDNA from samples collected from 8 to 61 days of age were analyzed for relative gene expression of *CTLA4*, *IL10* and *CXCL8*. Relative gene expression was determined by the efficiency-calibrated ΔΔCt method and is expressed as fold change relative to the calibrator sample. Gene expression profiles were obtained for individual chickens, using individual-specific day 29 samples as calibrators and 28S as a reference gene. Data were aligned and averaged by age (days). The overall effect of time was determined using a mixed effects regression model setting time and individual bird as a fixed and random effect respectively. When a significant time effect was found, post-hoc multiple means comparisons were made against the 8 d samples using the Tukey-Kramer p-value adjustment. Differences were considered significant at *P* < 0.05. Data are plotted as mean ± SEM; n = 5 chickens per time point.

Gene expression was conducted on cDNA prepared from samples collected between 8 and 61 days of age from growing feathers of non-expressing Brown line chickens. Of all genes measured, only *CTLA4*, *IL10* and *CXCL8* were found to change significantly with age (*P* = 0.0241, 0.005 and 0.0038 respectively; **Figure 5**; **Supplementary Table 5**). In contrast to Smyth chickens, individual variability was quite high and therefore no sample varied significantly from 8d samples.

### Association of overall changes in the TCR repertoire with infiltrating T cell subsets in growing feathers from Brown line chickens

To determine whether increases in overall changes in the T cell receptor repertoire (D-score) were associated with levels of T cells and their subsets in growing feather samples from non-expressing Brown line chickens, D-scores and pulp-infiltration data were tested for correlation by Spearman’s method (**Supplementary Table 6**). In growing feather samples from Brown line chickens, a highly significant negative correlation between the D-score of TCR-β2 and infiltrating CD4^+^CD8α^-^ T cells were found (*P* = 0.0001). No significant correlations between TCR-β1 D-score and infiltrating T cells were found in samples from Brown line chickens.

### Immunoregulatory activities in growing feathers from Brown line chickens

**Figure 6:**
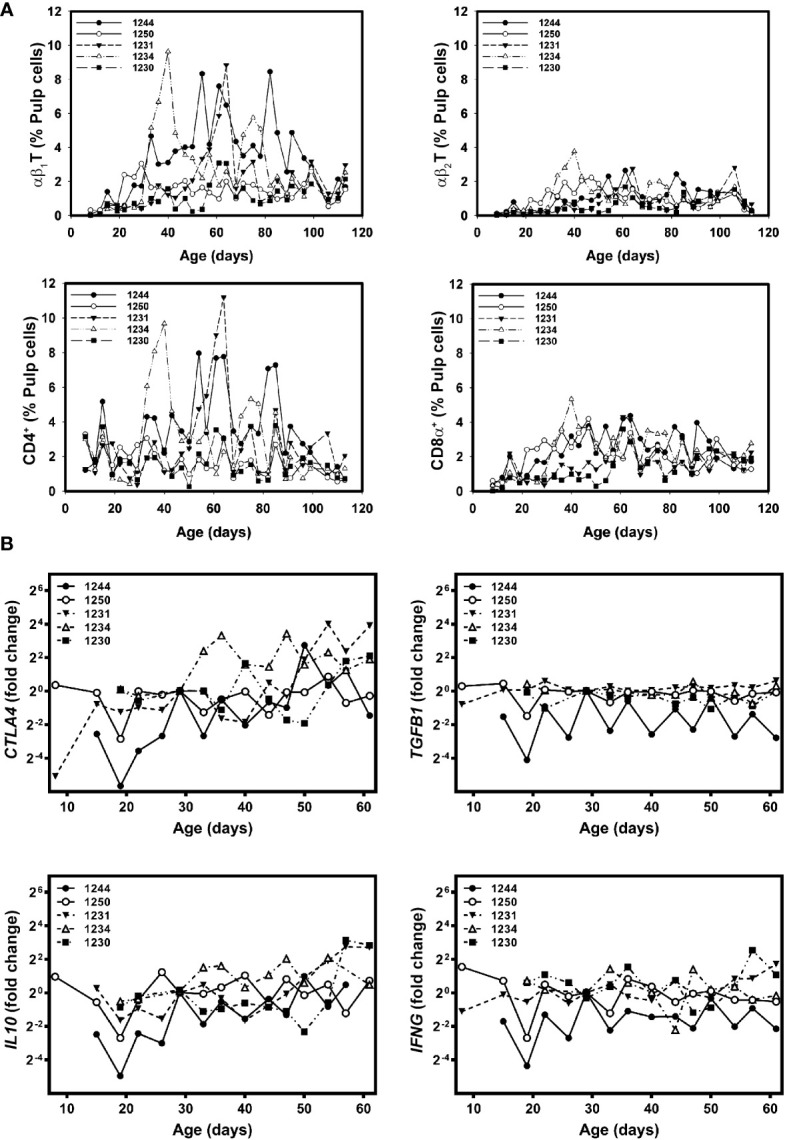
Individual-specific T cell subset infiltration and gene expressionprofiles in growing feathers of non-vitiligo expressing Brown line chickens. Growing feathers were sampled from Brown line chickens (n=5) beginning at 1-day post-hatch and continued twice a week for 113 days. **(A)** Single-cell suspensions were prepared from feather samples and immunofluorescently-stained for total leukocytes (CD45), B cells (Bu-1), γδ T cells (TCR1), αβ_1_ T cells (TCR2), αβ_2_ T cells (TCR3), CD4 and CD8α using chicken-specific antibodies. Population analysis was performed by flow cytometry. **(B)** RNA and cDNA fromfeather samples collected from 1 d to 61 d were prepared and used as templates for gene expression analysis with chicken-specific primers and probes. Relative gene expression was determined by the efficiency-calibrated ΔΔCt method and is expressed as fold change relative to the calibrator sample. Gene expression profiles were obtained for individual chickens, using individual-specific day 29 samples as calibrators and 28S as a reference gene.

To determine if significant differences found in growing feathers of Brown line chickens were the results of outliers, individual plots of cell infiltration and gene expression were examined (**Figure 6**). Three of the five chickens analyzed had substantial, yet transient rises in αβ_1_T and CD4^+^CD8α^-^ cells (1231, 1234 and 1244, **Figure 6A**). In the cell infiltration profiles of two of these three individuals (1231 and 1234) a small, transient increase in αβ_2_T and CD8α^+^ cells were also observed. None of the chickens with CD4^+^ cell infiltration showed any obvious changes in *IFNG* gene expression, however all showed substantial rises in *CTLA4* and *IL10* (**Figure 6B**).

### Foxp3 is expressed in growing feathers of vitiligo-expressing Smyth chickens and non-expressing Brown line chickens

**Figure 7:**
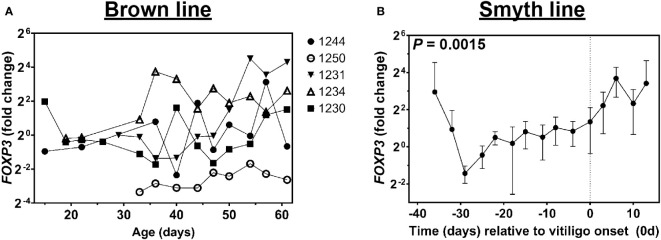
Expression of *FoxP3* in growing feathers of parental-control, non-vitiligo expressing Brown line chickens and vitiligo-expressing Smyth chickens. Growing feathers were sampled from Brown line **(A)** and Smyth **(B)** chickens beginning at 1-day post-hatch and continued twice a week for 113 days. **(A)** cDNA from samples collected from 8 to 61 days of age were analyzed for relative gene expression of *Foxp3*. Gene expression profiles were obtained for individual chickens, using individual-specific day 29 samples as calibrators and 28S as a reference gene. **(B)** cDNA from samples collected from 39 days prior to- through 13 days post-vitiligo onset were analyzed for relative gene expression of *FoxP3*. Data for vitiligo-expressing Smyth chickens were aligned by time (days) relative to age of vitiligo onset (set to 0 d and indicated by vertical dashed line). Smyth chickens were 43 to 57 days of age at time of vitiligo onset. The overall effect of time was determined using a mixed effects regression model setting time and individual bird as a fixed and random effect respectively. When a significant time effect was found, post-hoc multiple means comparisons were made against the -39 d and 8 d samples for Smyth and Brown line respectively, using the Tukey-Kramer p-value adjustment. Differences were considered significant at *P* < 0.05. Relative gene expression was determined by the efficiency-calibrated ΔΔCt method and is expressed as fold change relative to the calibrator sample. Data are plotted as mean ± SEM; n = 3 to 8 and 5 chickens per time point for Smyth and Brown line respectively.

In order to investigate the possible source of immunoregulatory genes in growing feathers of Smyth and Brown line chickens, we next tested for expression of *Foxp3* – the master transcription factor of regulatory T cells (35–37). Similar to *CTLA4* and *IL10*, three of five Brown line chickens analyzed had substantial, yet transient rises in *Foxp3* expression (1231, 1234 and 1244, **Figure 7A**). Interestingly, while a significant effect of age relative to vitiligo onset on *Foxp3* expression was detected in growing feathers of Smyth chickens (*P* = 0.0015), no significant increases were observed relative to -39d (**Figure 7B**).

## Discussion

The Smyth chicken represents a unique opportunity to examine the temporal, quantitative, and qualitative aspects of a variety of parameters in the evolving autoimmune vitiligo lesion. The anatomical location of the target tissue (the pulp of growing feathers) allows for repeated sampling of the same individual before and throughout disease development. This unique feature of the model enables the assessment of immunological activities before the emergence of symptoms which is essential to identifying predisposing factors. Moreover, the availability of the MHC-matched (*B* ^101/101^) genetically-susceptible but non-vitiligo-expressing, parental Brown line, from which the Smyth line was derived, presents unique opportunity for direct comparisons between non-expressing and expressing, vitiligo-susceptible individuals, that may prove valuable in identifying preventative mechanisms inherent in individual chickens. Using a combination of flow cytometry and mRNA expression analysis, we document a sequence of immunologically significant events leading up to and through progression of autoimmune vitiligo in the growing feather (GF) pulps of Smyth chickens. Using this approach, together with twice weekly collection of GF from day of hatch through 113 days of age, a much higher resolution of the leukocyte recruitment and activities in the target tissue, the GF pulp, was achieved compared to the study by Shi and Erf (28). In their study, GF samples from Smyth chickens were grouped as no vitiligo development, near vitiligo, active vitiligo and complete vitiligo, and analyzed by immunostaining of longitudinal GF-pulp sections. Never-the-less, key events like the steady infiltration of lymphocytes, including all TCR-, CD4-, and CD8-defined T cell subsets as well as B cells, starting two to three weeks before and reaching maximal levels around vitiligo onset were observed in both studies, attesting to the predictability of autoimmune activities during spontaneous vitiligo development in Smyth chickens. In the current study, inclusion of samples from Brown line chickens that did not develop vitiligo throughout the entire sampling period, revealed overall T and B cell presence in GF-pulps similar to those reported by Shi and Erf for Smyth chickens that did not develop vitiligo (29). Below, discussion will focus on integrating immune activities at the gene-expression- and cell recruitment/presence-levels as vitiligo develops and progresses in Smyth chickens along with events in GF-pulps of vitiligo susceptible, non-expressing Brown line chickens.

Surprisingly, the first indications of immunological activity in growing feathers of Smyth chickens appear to be regulatory in nature. A full month prior to vitiligo onset in GF-pulps of Smyth chickens, increases in the expression of the inhibitory receptor *CTLA4* were observed. These results mimic those seen in a 2020 immunohistochemistry study of tissue samples from patients with active vitiligo. Here, expression of CTLA-4 in lesional and marginal skin was significantly elevated compared to non-lesional skin (38). In competition with the co-stimulatory CD28 receptor expressed on conventional T cells, CTLA-4 binds the co-stimulatory ligands B7-1 (CD80) and B7-2 (CD86) on the surface of antigen-presenting cells thereby inhibiting T cell activation (39). *CTLA4* is constitutively expressed in CD4^+^ regulatory T cells (Tregs) which can be identified by expression of the transcription factor *FOXP3*. In addition to Tregs, *CTLA*4 is also induced in conventional T cells early in activation (40, 41). While we observed expression of *FOXP3* at these early time points the trend of *CTLA4* expression clearly follows infiltration of both CD4^+^ and CD8^+^ αβT cells into the target tissue, suggesting the increased CTLA4 expression is associated with early events in antigen-specific activation of conventional T cells. Moreover a 2012 report documented a negative correlation between cytotoxic T cells and Tregs in the blood of patients with generalized vitiligo (42). Unfortunately, antibodies for chicken Foxp3 are currently lacking and available markers for Tregs, such as CD25, are also associated with activation of conventional T cells.

In addition to the expression of immunoregulatory genes prior to vitiligo onset in Smyth chickens, we observed a steady increase in *IL21R* expression, the receptor for IL-21. In an IL-21R-deficient non-obese diabetic (NOD) mouse model for autoimmune (type 1) diabetes, IL21 signaling was demonstrated to be indispensable for disease onset (43). Furthermore, in a virus-induced mouse model for type-1 diabetes on the NOD background, a dramatic reduction in diabetes incidence in IL-21R-deficient mice was reversed with the addition of IL-21 receptor sufficient dendritic cells. The authors noted the failure of IL-21R deficient dendritic cells to acquire CCR7 which is needed for their migration to lymph nodes, may explain the reduction in the diabetes onset (44). Curiously, while IL-21 was detected in our study, its levels in the target tissue did not increase until much closer to vitiligo onset.

The first signs of leukocyte recruitment activities were seen much closer to disease onset. Notably, increases in transcription of T cell recruitment molecules (*CCL19, CCR7*) correlated with increased expression of cytokines (*IL1B, IL18, IL10, IL21*) and appear to precede T cell infiltration into the target tissue further suggesting local tissue activities triggering development of autoimmune vitiligo. Local activation of tissue-resident T cells may account for increases in *IL2R* expression which is induced on activated T cells in order to respond to IL-2 enabling their proliferation. Interestingly, alteration of the TCR-β_2_ repertoire, without significant increases in αβ_2_ T cell levels, was also observed further implying local stimulation of T cells. Elevation of *IL21* further argues for T cell activation in the target tissue at this stage of vitiligo development. In addition to well established roles in sustaining the effector phenotype of activated T cells (45, 46), IL-21 is also suspected to play a role in the formation of memory T cells (47, 48), B cell activation and plasma cell generation (49). Also interesting is the upregulation of the potent anti-inflammatory cytokine *IL10* which may be induced in activated T cells, including Tregs (50) as well as B cells (51).

While vitiligo is generally accepted to be an autoimmune-driven disorder, the role of B cells and T cells in the initiation of the disease is still unknown. In vitiligo expressing Smyth chickens, the earliest rises of infiltrating leukocytes were seen in the levels of αβ_1_T cells 11 days prior to visual onset, clearly preceding the first significant increase in B cells. Rises in αβ_1_T cells were accompanied by increases in *IFNG* which is thought to play a central role in driving the progression of autoimmune vitiligo (29, 52, 53). Eight to four days prior to visual onset saw the further development of the autoimmune response with significant increases CD8α*^+^* cells which are likely cytotoxic T cells based on the simultaneous increases in *FASLG* and *GZMA* expression. Also increased were αβ_2_T and CD4*^+^* CD8α^+^ cells. While CD4^+^CD8α^+^ lymphocytes are a relatively minor component of the T cell compartment, the emergence of additional cells CD4^+^CD8^+^ in the active lesion is notable. Chicken-specific studies suggest that these cells are of a CD4^+^ T cell origin (54) and that their presence in the periphery increases with age (Erf et al., 1998) (55). Recent data from mammals suggest that CD4^+^CD8α^+^ are antigen-experienced and possibly of CD8-origin (56, 57).

While B cell levels were the dominant population in terms of total proportions of CD45^+^ cells, they were not significantly increased until visual onset of vitiligo (0d). The late entry of B cells may explain the notion of the generation of autoantibodies as a consequence and not a cause of melanocyte destruction in vitiligo. It should be noted that “onset” was defined by the depigmentation, and hence melanocyte loss, at the most proximal end of the feather pulp where the epidermal melanocyte-containing barb ridge is located.

The “peak” of the response in terms of levels of immune activity occurred 3-6 days post visual onset. Here, maximum levels of nearly every measured parameter were reached. However, post-onset, B cells, CD4^+^CD8α^+^ and CD4^+^CD8α^-^ cells eventually returned to basal levels whereas CD4^-^CD8^+^ T cells maintained an elevated presence in the feather pulp. As described previously, at this stage these cells were in the barb ridge in close association with apoptotic melanocytes, similar to observations reported in human vitiligo skin (26, 58, 59). Additionally, elevated levels of *CCL19*, *CCR7*, *CTLA4*, *CXCL8*, *FASLG*, *GZMA*, *IFNG*, *IL1B*, *IL2R*, *IL10*, *IL18*, *IL21* and *IL21R* were also maintained in the tissue until at least 13 days post onset (the last time point measured) while those of *TGFB1* returned to basal levels six days post onset.

Using a low resolution but fully comprehensive approach to monitor the T cell repertoire, we were able to detect positive correlations between infiltrating T cells and changes in their T cell receptor diversity which is indicative of clonal expansion. We were also able to correlate changes in the repertoire with the increased frequency of specific allele groups, reiterating the notion of a clonal shift in T cell diversity in the target tissue. In agreement with past studies, the T cell infiltrate was dominated by αβ_1_ T (TCR2^+^) cells (60). It is likely that the relatively larger numbers of TCR2^+^ cells (vs. TCR3^+^ αβ_2_ T cells) in the tissue contributed to the inability to detect an overall effect of time on T cell diversity relative to vitiligo onset. It should be noted that the number of TCR Vβ-gene families is relatively small in chickens compared to humans (2 vs. 52) and therefore the ability of AFLP to detect changes is likely diminished. Additionally, though early samples were chosen as references for comparison, their TCR-length distribution patterns were not normal (i.e. Gaussian) which implies that the pool of TCRs in the tissue may already be skewed relative to a pool of naïve T cells from the periphery. A repeat study utilizing naïve T cells from the periphery would likely provide a cleaner measurement of the alterations in diversity.

The parental-control Brown line, from which the Smyth chicken was derived, has long been known to be susceptible to vitiligo, with 0 to 2% of individuals in each flock expressing the disorder. The strongest evidence for the inherent susceptibility for autoimmune vitiligo within the Brown line was however contributed by an observed greater than 70% incidence following administration of DNA methylation inhibitor 5-azacytidine (18). The current study is the first report to suggest a role for immunoregulatory mechanisms, specifically Treg activity, in preventing disease onset in this line of chickens. In a subgroup of non-expressing Brown line chickens, transient increases in *FOXP3*, *CTLA4* and *IL10* expression were observed and were correlated with infiltration of CD4^+^ T cells. In contrast to Smyth chickens, Tregs in this subgroup of Brown line chickens are likely to have succeeded in preventing the initiation of the immune response, especially as their expression was not followed or accompanied by increases in *IFNG* and *IL21* as seen in Smyth chickens.

While we hypothesize that expression of *CTLA4* is driven by distinct cell populations in Smyth and Brown line chickens, it is possible that a defect in CTLA-4 may be playing a role in permitting and preventing development of vitiligo. In the non-obese diabetic mouse model, alternative isoforms of *CTLA4* (e.g. soluble, B7-independent) have been identified and are suspected to have a role in type 1 diabetes onset through impaired Treg function (61–65). Polymorphisms in *CTLA4* have been identified in vitiligo patients although their association with susceptibility is controversial (66–71). Meta-analysis of published studies suggest that impacts on susceptibility may be allele-dependent or not specific to vitiligo but to autoimmunity in general (72–74).

If the immunosuppressive roles of *CTLA4* and *IL10* in Brown line chickens are confirmed, this would imply a common melanocyte-specific triggering of a T cell-driven immune response in both vitiligo-prone Smyth and -susceptible Brown line chickens with the former progressing to a cell-mediated autoimmune response and the later to an immunoregulatory response, perhaps resulting in the establishment of peripheral tolerance (anergy). Future studies should work to test this hypothesis as it would represent a clear paradigm shift for the Smyth chicken model and warrant its further development as a model for the study of autoimmune vitiligo.

Here we have collected a substantial amount of data, however, the study was limited by the available reagents and tools for avian immunology studies and the need to base some of the interpretations on transcriptome data and correlations, Nevertheless, the information presented offers unique insights into events in the vitiligo target tissue that lead to full or no disease expression in chickens with similar susceptibility and genetic background. Hence, future focus on epigenetic mechanisms underlying loss or maintenance of tolerance in the Smyth versus Brown line chickens, may identify critically important mechanisms in the prevention and re-establishment of tolerance in autoimmune vitiligo.

## Data availability statement

The raw data supporting the conclusions of this article will be made available by the authors, without undue reservation.

## Ethics statement

The animal study was approved by University of Arkansas, Fayetteville, Institutional Animal Care and Use Committee. The study was conducted in accordance with the local legislation and institutional requirements.

## Author contributions

DF: Conceptualization, Data curation, Formal analysis, Investigation, Methodology, Validation, Visualization, Writing – original draft, Writing – review & editing. KB: Writing – review & editing, Formal analysis, Methodology. MS: Formal analysis, Writing – review & editing. GE: Conceptualization, Funding acquisition, Methodology, Project administration, Resources, Software, Supervision, Writing – original draft, Writing – review & editing.
