# Supplementary material for: Spontaneous immunological activities in the target tissue of vitiligo-prone Smyth and vitiligo-susceptible Brown lines of chicken
Source: Front Immunol. 2024 Apr 24;15:1386727. doi: 10.3389/fimmu.2024.1386727 (PMC11076693; doi:10.3389/fimmu.2024.1386727)
Supplement: Supplementary file 1 [file DataSheet_1.pdf]

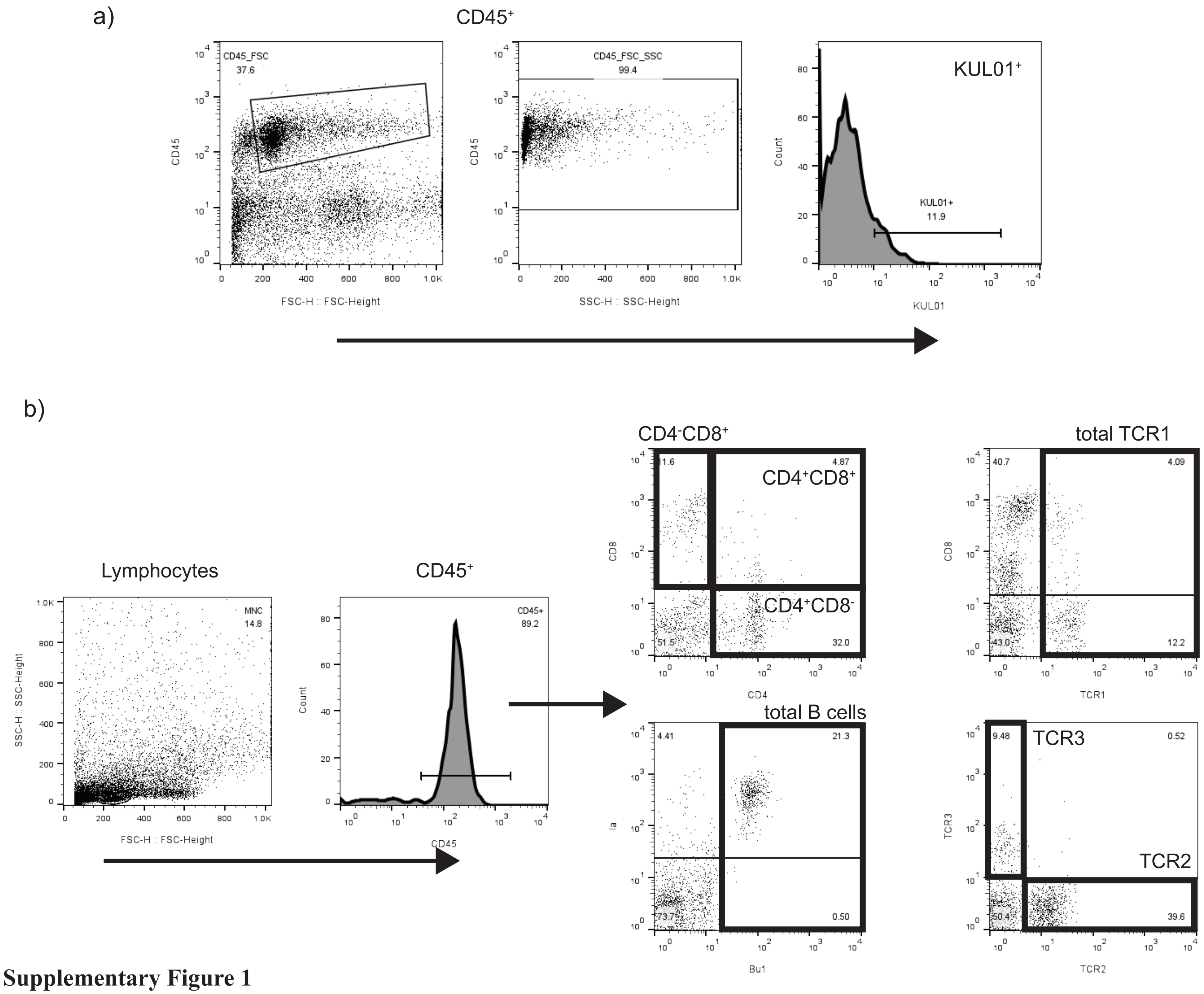


**Supplementary Figure 1**

**Gating strategy used for flow cytometry analysis.** a) All CD45$^{+}$ cells were analyzed for expression of the chicken macrophage marker KUL01. b) In separate stainings CD45+ lymphocytes were analyzed for expression of CD4 and CD8α (top left), TCR1 (top right), chicken B cell marker Bu1 (bottom left) and TCR2 and TCR3 (bottom right).
