# Supplementary material for: Spontaneous immunological activities in the target tissue of vitiligo-prone Smyth and vitiligo-susceptible Brown lines of chicken
Source: Front Immunol. 2024 Apr 24;15:1386727. doi: 10.3389/fimmu.2024.1386727 (PMC11076693; doi:10.3389/fimmu.2024.1386727)
Supplement: Supplementary file 2 [file Table_1.docx]

**Supplemental Table 1**

**Monoclonal antibody panels used for immunofluorescent-staining^1^**

| Panel | Target | Fluorophore |
| --- | --- | --- |
| Macrophages | CD45  KUL01 | SPRD |
|  |  | rPE |
| T cells | CD45  CD4  CD8α | SPRD |
|  |  | FITC |
|  |  | rPE |
| B cells | CD45  Bu1  Ia | SPRD |
|  |  | FITC |
|  |  | rPE |
| γδT cells | CD45  TCR1  CD8α | SPRD |
|  |  | FITC |
|  |  | rPE |
| αβT cells | CD45 | SPRD |
|  | TCR2 | FITC |
|  | TCR3 | rPE |

^1^ All antibodies were purchased from SouthernBiotech, Birmingham, AL

**Supplemental Table 2**

**Target gene primer^1^ and probe^2^ sequences**

| Target | Accession NO. | Primer/Probe | Sequence (5’-3’) |
| --- | --- | --- | --- |
| 28S^3^ | X59733 | Forward | GGCGAAGCCAGAGGAAACT |
|  |  | Reverse | GACGACCGATTTGCACGTC |
|  |  | Probe | AGGACCGCTACGGACCTCCACCA |
| CCL19^8^ | NM_001302168.1 | Forward | GCTGCGCCTCCGAGAGA |
|  |  | Reverse | ACACTTCTGCAGAGCCTAATTGC |
|  |  | Probe | CAGCTCTGCCAGGAAGGTCCCAAATC |
| CCR7^8^ | NM_001198752.1 | Forward | ACCATGGACGGCGGTAAA |
|  |  | Reverse | ATGGTGGTGTTGGCGTCATA |
|  |  | Probe | TGTGCTGGGAACAACGTCACCGAC |
| CTLA4^8^ | NM_001040091.1 | Forward | GGGTCACCGTGAGCTTTCTC |
|  |  | Reverse | GCCTGTTGGCCAGTACTATTGC |
|  |  | Probe | CGCAGCCACCGCCACTGC |
| FASLG^8^ | NM_001031559.1 | Forward | CCAGTGAAAAAGGAAGCAAGGA |
|  |  | Reverse | GAGACAGGTTCCCACTCCAATG |
|  |  | Probe | CAGCACACTTAACAGGAAACCCCACACAG |
| GZMA^8^ | NM_204457.1 | Forward | CAGCTGCTCATTGCAATCTGA |
|  |  | Reverse | GGACAGTAGTCTGGGTAGCGAATT |
|  |  | Probe | CAGAGTTATTCTTGGAGCCCATTCACGGAC |
| IFNA3^6^  (IFN-α, IFNA) | NM_205427.1 | Forward | GACAGCCAACGCCAAAGC |
|  |  | Reverse | GTCGCTGCTGTCCAAGCATT |
|  |  | Probe | CTCAACCGGATCCACCGCTACACC |
| IFNW1^6^  (IFN-β, IFNB) | NM_001024836.1 | Forward | CCTCCAACACCTCTTCAACATG |
|  |  | Reverse | TGGCGTGTGCGGTCAAT |
|  |  | Probe | TTAGCAGCCCACACACTCCAAAACACTG |
| IFNG^3^ | NM_205149.1 | Forward | GTGAAGAAGGTGAAAGATATCATGGA |
|  |  | Reverse | GCTTTGCGCTGGATTCTCA |
|  |  | Probe | TGGCCAAGCTCCCGATGAACGA |
| IL1B^3^ | NM_204524.1 | Forward | GCTCTACATGTCGTGTGTGATGAG |
|  |  | Reverse | TGTCGATGTCCCGCATGA |
|  |  | Probe | CCACACTGCAGCTGGAGGAAGCC |
| IL8L2^5^  (IL8, CXCL8) | NM_205498.1 | Forward | GCCCTCCTCCTGGTTTCAG |
|  |  | Reverse | TGGCACCGCAGCTCATT |
|  |  | Probe | CTTTACCAGCGTCCTACCTTGCGACA |
| IL10^4^ | NM_001004414.2 | Forward | CATGCTGCTGGGCCTGAA |
|  |  | Reverse | CGTCTCCTTGATCTGCTTGATG |
|  |  | Probe | CGACGATGCGGCGCTGTCA |
| IL18^8^ | NM_204608.1 | Forward | GGCAGTGGAATGTACTTCGACAT |
|  |  | Reverse | ACCTGGACGCTGAATGCAA |
|  |  | Probe | ACTGTTACAAAACCACCGCGCCTTCA |
| IL21^7^ | NM_001024835.1 | Forward | GTGGTGAAAGATAAGGATGTCGAA |
|  |  | Reverse | TGCCATTCTGGAAGCAGGTT |
|  |  | Probe | TGCTGCATACACCAGAAAACCCTGGG |
| IL2RA^8^ (CD25) | NM_204596.1 | Forward | GCCAGCAAGACAAACCCAAA |
|  |  | Reverse | GGCATACCGCAAAAACTTGAA |
|  |  | Probe | CCCAGCACCTCCGAAGCAAGCA |
| IL21R^8^ | NM_001030640.1 | Forward | CAGTACTCCACGTGTCACGAAAA |
|  |  | Reverse | TGGCACCCAGGTCATTCCT |
|  |  | Probe | ACTATGTGCAGACCCTGTCGTGCCTCC |
| TGFB1^8^ | NM_001318456.1 | Forward | GGTTATATGGCCAACTTCTGCAT |
|  |  | Reverse | CCCCGGGTTGTGTTGGT |
|  |  | Probe | AGCGCCGACACGCAGTACACCA |
| FOXP3^9^ | MT133687.1 | Forward | AGTACGCCACAACCTGAGCCT |
|  |  | Reverse | TTGGGGTCCTCTCAGCTCCGT |
|  |  | Probe | TGCGGGTGGAGAACGTACGTGGG |

^1^ All oligos were synthesized by Eurofins Genomics, Louisville, KY

^2^ Probes were labeled with FAM and TAMARA on the 5’- and 3’-ends respectively

^3, 4, 5, 6, 7^ Sequences from Kaiser *et al*., 2003; Rothwell *et al.*, 2004; Kogut *et al.*, 2005; He *et al.*, 2012 and Shi and Erf, 2012, respectively

^8^ Primers and probes were designed using Primer Express 3.0 (Applied Biosystems, Foster City, CA.)

^9^Sequences from Burkhardt *et al.*, 2022

**Supplemental Table 3.**

**Primers^1^ used for T cell receptor spectratyping**

| Gene segment | Accession NO. | Primers | Sequence (5’-3’) |
| --- | --- | --- | --- |
| TCR Variable-β_1_ | EF554743.1  EF554744.1  EF554745.1  EF554746.1  EF554747.1  EF554748.1  EF554749.1  EF554750.1  EF554751.1  EF554752.1  EF554753.1  EF554754.1  EF554755.1  EF554756.1  EF554757.1  M81147.1  M37802.1  M37803.1  M37804.1  M37805.1 | Forward^2^ | GTGGGACTAAGGAGAAATCC |
| TCR Variable-β_2_ | EF554759.1 EF554760.1 EF554761.1 EF554763.1 EF554764.1 EF554765.1 EF554768.1 EF554769.1 EF554772.1 EF554773.1 EF554774.1 EF554775.1 EF554776.1 EF554777.1 EF554778.1 EF554779.1 EF554780.1 EF554782.1  M37806.1  M81149.1  M81148.1  M81150.1  M81151.1 | Forward^3^ | GCGATATTCACACCGGATAC |
| TCR Constant-β | EF554742.1 | Reverse | GATCAGGGAAGAAACCAGAG |

^1^ Oligos were synthesized by Applied Biosystems, Foster City, CA

^2^ Contains a 5’-VIC label

^3^ Contains a 5’-FAM label

**Supplemental Table 4**

**Spearman correlation coefficients relating perturbations in T cell repertoire (D-score) with infiltrating T cell and T cell subsets in growing feathers of vitiligo-expressing Smyth chickens.**

| Line | D-score | T cell | Spearman’s  correlation  coefficient (ρ) | P-value^1^ |
| --- | --- | --- | --- | --- |
| Smyth | TCR-β_1_ | αβ_1_ T | 0.1093 | 0.2494 |
|  |  | CD4^+^ | -0.0035 | 0.9710 |
|  |  | CD8α^+^ | 0.0900 | 0.3430 |
|  |  | CD4^+^CD8α^+^ | 0.1031 | 0.2772 |
|  | TCR-β_2_ | αβ_2_ T | 0.4064 | <0.0001* |
|  |  | CD4^+^ | -0.0240 | 0.8008 |
|  |  | CD8α^+^ | 0.4350 | <0.0001* |
|  |  | CD4^+^CD8α^+^ | 0.3353 | 0.0003* |

^1^P-values <0.05 were considered statistically significant (*)

**Supplemental Table 5**

**Genes with no overall changes in expression relative to age in growing feathers of parental-control, non-expressing Brown line chickens**

| Gene | P-value |
| --- | --- |
| *CCL19* | 0.1518 |
| *CCR7* | 0.3619 |
| *FASLG* | 0.6281 |
| *GZMA* | 0.6149 |
| *INFA* | 0.6772 |
| *IFNB* | 0.4729 |
| *IFNG* | 0.6055 |
| *IL1B* | 0.6360 |
| *IL2R* | 0.0864 |
| *IL18* | 0.7660 |
| *IL21* | 0.3447 |
| *IL21R* | 0.2106 |
| *TGFB1* | 0.6912 |

**Supplemental Table 6**

**Spearman correlation coefficients relating perturbations in T cell repertoire (D-score) with infiltrating T cell and T cell subsets in growing feathers of non-expressing Brown line chickens.**

| Line | D-score | T cell | Spearman’s  correlation  coefficient (ρ) | P-value^1^ |
| --- | --- | --- | --- | --- |
| Brown | TCR-β_1_ | αβ_1_ T | 0.0394 | 0.7498 |
|  |  | CD4^+^ | -0.0836 | 0.4979 |
|  |  | CD8α^+^ | 0.0880 | 0.4757 |
|  |  | CD4^+^CD8α^+^ | -0.0425 | 0.7306 |
|  | TCR-β_2_ | αβ_2_ T | 0.1079 | 0.3811 |
|  |  | CD4^+^ | -0.4514 | 0.0001* |
|  |  | CD8α^+^ | 0.0047 | 0.9696 |
|  |  | CD4^+^CD8α^+^ | -0.1724 | 0.1598 |

^1^P-values <0.05 were considered statistically significant (*)
